# Muscularity and Strength Affect Individual Variation in Self-Perception of Fighting Ability in Men

**DOI:** 10.3389/fpsyg.2019.00018

**Published:** 2019-01-22

**Authors:** José Antonio Muñoz-Reyes, Pablo Polo, Carlos Rodríguez-Sickert, Paula Pavez, Nohelia Valenzuela, Oriana Ramírez-Herrera

**Affiliations:** ^1^Laboratorio de Comportamiento Animal y Humano, Centro de Estudios Avanzados, Universidad de Playa Ancha, Valparaíso, Chile; ^2^Grupo UCM de Estudio del Comportamiento Animal y Humano, Departamento de Psicobiología, Facultad de Psicología, Universidad Complutense de Madrid, Madrid, Spain; ^3^Centro de Investigación en Complejidad Social, Facultad de Gobierno, Universidad del Desarrollo, Santiago, Chile; ^4^Departamento de Ciencias Biológicas, Facultad de Ciencias Biológicas, Universidad Andrés Bello, Viña del Mar, Chile

**Keywords:** fighting ability, hand grip strength, muscularity, men, self-perception

## Abstract

**Objective:** There is evidence that competitive conflicts are the main form of intrasexual competition among men. The capacity to recognize visual cues of fighting ability in competitors is thought to be an important characteristic that allows men to avoid the costs of contest competition. However, for an accurate comparison to take place, individuals need to compare the fighting ability of their competitors to their own to assess this asymmetry.

**Methods:** In order to improve our understanding of this self-assessment process, here we study the relationship between visual fighting ability cues, namely (i) muscularity, as measured with a bioimpedance device, (ii) the real capacity to inflict cost to a rival based on strength, as measured with a hand grip dynamometer (HGS), and (iii) self-perceived fighting ability, as determined with a questionnaire. The study sample was 364 men between 18 and 38 years of age (*M* ± *SD* = 22.27 ± 3.99).

**Results:** Our results confirm the expected positive relationship between upper-body muscularity and strength, while controlling for body mass index (BMI). However, muscularity explained only around 30.2% of the variance in strength. In addition, muscularity was related to self-perception of fighting ability in our sample, its effect being partially mediated by strength.

**Conclusion:** The more muscular men perceive their fighting ability as being greater, and not only because they are stronger (at least in the HGS task). Accordingly, it seems that men take into account the overestimation the robustness of the relationship between strength and muscularity that prevails within his peers.

## Introduction

The visual signs of physical strength, such as body muscularity, are crucial to understanding how men navigate in social environments (Puts et al., 2015). This is especially true in same-sex competitive interactions, where physical signs of strength are thought to be relevant cues of fighting ability that allow for resolving these conflicts in favor of the stronger man (Sell et al., 2012). In thisway, conflicts are usually resolved by intimidation strategies, such as the use of anger, which avoid the costs associated with physical aggression (Sell et al., 2009b). Previous studies have found clear relationships between direct measurements of strength, such as hand grip strength (HGS), and the self-perception of fighting ability (e.g., Muñoz-Reyes et al., 2015). However, strength *per se* is not a visual cue of fighting ability that can be appreciated by rivals, and there are currently no studies about the direct relationship between an objective measurement of muscularity, a visual sign, and the self-perception of fighting ability.

Since the study of Wilson and Daly (1985) about the young male syndrome, which applied an evolutionary perspective to explain the willingness of men to participate in risky or violent competitive interactions, many studies have inquired into the functional value of direct aggression as a mechanism of intrasexual competition (e.g., Buss and Shackelford, 1997; Archer, 2009; Goetz, 2010; de Bruyn et al., 2012; Liddle et al., 2012; McDonald et al., 2012). This pattern of behavior is expected in men since, as a general tendency among mammals, males invest less energy in reproduction, in concordance with standard parental investment model, becoming the more competitive sex (Bateman, 1948; Trivers, 1972). In this sense, evidence indicates that men make more frequent and intense use of physical aggression than do women (e.g., Archer, 2009; Muñoz-Reyes et al., 2012), and that competitive conflict is the main form of intrasexual competition among men (Navarrete et al., 2010). Therefore, intrasexual competition through the use of aggressive repertoires is probably one of the most powerful selective evolutionary forces that modeled men’s bodies and minds (see Sell et al., 2009a, 2012; Puts, 2010). This selective pressure has produced clear sexual dimorphism in strength (Kirchengast, 2010; Isen et al., 2014), especially for the upper body, where men are generally 90% stronger than women (Murray et al., 1985; Bohannon, 1997; Stoll et al., 2000). A dimorphic characteristic that increases attractiveness in men (Frederick and Haselton, 2007; Hönekopp et al., 2007; Crossley et al., 2012; Sell et al., 2017a), correlates positively with their mate value (Muñoz-Reyes et al., 2015), and is considered as a crucial trait driving differences in the ability to inflict cost to a rival in unarmed or armed combat (Sell et al., 2012).

However, not all men express aggression with the same frequency and intensity (Sell et al., 2009b, 2016, 2017b; Muñoz-Reyes et al., 2012). The common explanation for these major differences is based on the evolutionary concept of resource holding power (RHP), which evolutionary psychologists apply to our species (Sell et al., 2009b). This is a mechanism to assess asymmetries in the fighting abilities of contestants (Parker, 1974) i.e., the faculty to assess fighting ability in a rival based on physical traits, and thus compare the difference in fighting ability between contestants. This gives men the possibility of deciding to fight or flee when faced with a conflict, a mechanism that is adaptive, and leads to a positive and close relationship between the development of physical signs of strength as cues of fighting ability and the propensity to deploy direct physical aggression, which in turn explains individual differences in aggressiveness. Accordingly, several studies (Sell et al., 2009b, 2010, 2012, 2016, 2017a,b) have demonstrated that both sexes, but especially men, can assess fighting ability based on formidable traits like physical signs of strength, upper-body muscularity being the most robust trait (Durkee et al., 2018). This ability gives men a social tool to regulate conflict. These results indicate the relevance of physical signs of strength for men in contest competition, this being an important trait to understand male behavior, especially in the deployment of aggressive intrasexual competition tactics. In fact, recent studies indicate that more formidable men believe more in the use and utility of warfare (Sell et al., 2017b), have a lower threshold for the use of aggression, especially anger (Sell et al., 2009b), and feel that they deserve better social outcomes (Sell et al., 2016). The logical argument above highlights the relevance of assessing muscularity as a reliable manner to estimate strength, which in turn is the key trait to win fights and extract limited resources from social and natural environments.

In order to fully understand the role of visual cues of physical strength such as upper body muscularity on aggressive deployment and the resolution of conflicts, we need to study not only its use by contestants as proxies for physical strength and fighting ability of their opponents, but also to study how the individual’s own visual cues of physical strength and his real physical strength inform his self-perception of his fighting ability. As in any signaling model of conflict/bargaining with incomplete information, it is the relative comparison between the former and the latter what should inform behavior in intra-sex male competitive interactions (Sanchez-Pages, 2012). However, little is known about the real superposition between the two variables and the role of each in explaining the self-perception of fighting ability, especially, when individuals assess their strength without relying on physical cues.

Therefore, in this study, we assess the relevance of muscularity, and specifically upper-body muscularity, to understanding self-perception of fighting ability. To do this, we have applied the Self-Perceived Fighting Ability Questionnaire (Muñoz-Reyes et al., 2012) to a sample of 364 young men, which has been demonstrated to be reliably associated with hand-grip strength and aggression (Muñoz-Reyes et al., 2012, 2015). We investigated the relationship between self-perceived fighting ability and two objective anthropometric variables, strength (as measured by a hand-grip dynamometer), and upper-body muscularity (as determined by a body composition bioelectrical impedance device).

First, we expect upper-body muscularity to be a good predictor of physical strength. Males ability to accurately assess strength from physical visual cues was an adaptive trait in the context of intra-sexual competition. Strength in itself can be relevant in competitive physical contests, but to the extent that strength is a key feature of fighting ability (Sell et al., 2009a), assessing one opponent’s strength from visual cues becomes even more important for males to regulate conflict and navigate their social environment.

Second, assessing one’s own strength regardless of physical cues is easier than assessing an unknown rival’s strength, without relying on physical cues. Thus, if strength is the key feature of fighting ability (Sell et al., 2009a), we expect that strength will mediate the relationship between upper-body muscularity and self-perception of fighting ability.

## Materials and Methods

### Participants

The sample was composed of 363 men between 18 and 38 years old (*M* ± *SD* = 22.29 ± 3.98). The sample was drawn through public announcements in the 5th Region of Valparaíso, Chile. Most of the participants were middle-class university students.

### Ethics Statement

The Bioethic Committee of the University of Playa Ancha and the Ethic Committee of FONDECYT authorized the research. A written informed consent was obtained from the participants of this study. Data were anonymized, and all written informed consent have been kept in the Laboratorio de comportamiento animal y humano ^1^.

### Self-Perception Measures

#### The Self-Perceived Fighting Ability Questionnaire

We applied a validated Chilean version of the Self-Perceived Fighting Ability Questionnaire (Muñoz-Reyes et al., 2012), which had been applied previously in Chile (see Muñoz-Reyes et al., 2015). The questionnaire is composed of four questions (1—How good of a fighter am I? 2—What is the perception that others have of my skills as a fighter? 3—How much fear can I provoke in someone who is about to fight with me? 4—What chance would I have of winning a fight if I had to fight with someone?). Participants answered according to their self-perceived position in the social group based on a seven-point Likert scale (i.e., 1 “well below average” to 7 “well above average”). The Cronbach alpha was α = 0.82, which was slightly higher than that of an earlier study in Chile (α = 0.78 in Muñoz-Reyes et al., 2015).

### Anthropometric Measures

#### Upper Body Strength

We used a hydraulic hand grip dynamometer (Jamar^®^ 5030J1) to assess upper-body strength. The protocol for data collection has been applied previously in other research (see Gallup et al., 2007; Muñoz-Reyes et al., 2012). We registered three measurements for each hand, with a one-minute rest between each strength test. The highest HGS scores were used for analysis (Muñoz-Reyes et al., 2012).

#### Upper Body Muscularity

We first measured the participants’ height, which was obtained barefoot with a manual stadiometer, and registered in centimeters (SECA^®^ 203). An InBody 370 body^®^ composition analyzer was used to measure upper-body muscularity in kilograms. The InBody^®^ 370 employs a tetrapolar 8-point tactile electrode (2 for each foot and hand). The methods consist of the passage of imperceptible low-amplitude electric currents that measure resistance and reactance values (i.e., bioelectrical impedance) to estimate body composition (Cunha et al., 2018). The machine treats the body as five cylinders composed of the four limbs and the trunk, and measures the bioelectrical impedance of these parts separately. Three frequencies (5, 50, and 250 kHz) were employed to measure impedance in the five segments. Unlike conventional bioelectrical impedance, this analysis does not rely on formulas to estimate whole body composition. This technique has been reported to be a valid tool for assessing total and segmental body composition (Ling et al., 2011; Bosy-Westphal et al., 2017). Among the data recorded by the device was fat-free mass measurements of the trunk, and right and left arms, which were added together to obtain an estimation of upper-body muscularity. Although the device registers total skeletal muscle mass, only the fat-free mass was available in the segmental analysis. However, total fat-free mass and muscular mass correlated strongly in our sample (*r* = 0.984, *N* = 363, *p* < 0.001). In addition, we collected data on the participants’ body mass index (BMI).

### Statistical Analyses

To test the first prediction, that upper-body muscularity is a good predictor of strength, we performed a simple linear regression analysis between HGS and the measurement of upper-body muscularity as the predictor variable (i.e., upper-body fat-free mass). Following previous research (Lassek and Gaulin, 2009), we used BMI as a covariable in all tested models of the predictions, which allowed us to take into account that some muscle mass supports body fat mass. Age was also considered as a covariable in all tested models of the predictions as muscularity and strength may change over time.

To test the second prediction, we run a linear regression analysis of self-perceived fighting ability as the outcome variable and upper-body muscularity as the predictor variable, with BMI and age as covariables. Finally, we estimate the mediating effect of strength on the relationship between fighting ability and upper-body muscularity. We tested the mediation analyses with a bootstrapping method (5000 bootstraps and *p* = 95%) using PROCESS macro for SPSS^2^. This technique is used to establish the direct and indirect (mediation) effect of an established relationship between variable A (muscularity) on variable B (perceived fighting ability). All the analyses were performed with IBM SPSS software, with a level of significance set at α = 0.05.

## Results

**FIGURE 1:**
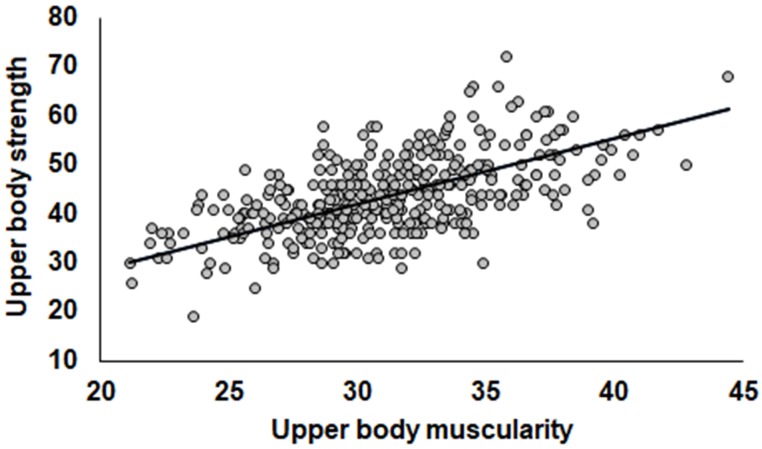
Relationship between upper body muscularity and strength statistically controlling for BMI.

For the first prediction, our linear regression model showed a positive relationship between upper body muscularity and strength while controlling for BMI and age [*R*^2^ = 0.36, *F*_(3,359)_ = 68.878, *p* < 0.001]. This result indicated that more muscular men are stronger (*B* = 1.345, *SE* = 0.104, β = 0.683, *p* < 0.001; see Figure 1).

**FIGURE 2:**
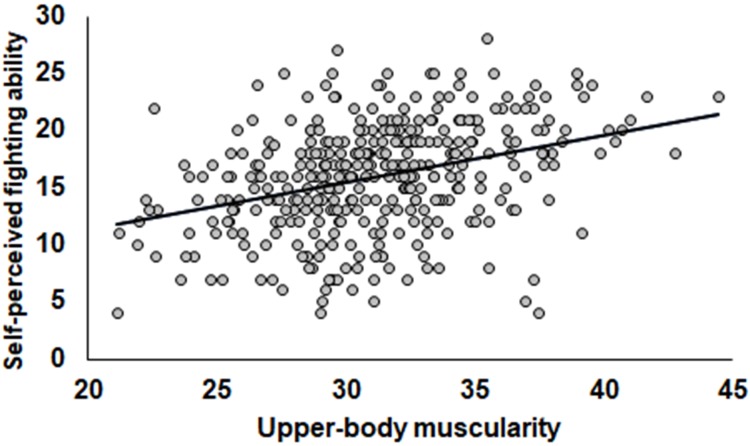
Relationship between upper body muscularity and self-perceived fighting ability controlling for BMI.

**FIGURE 3:**
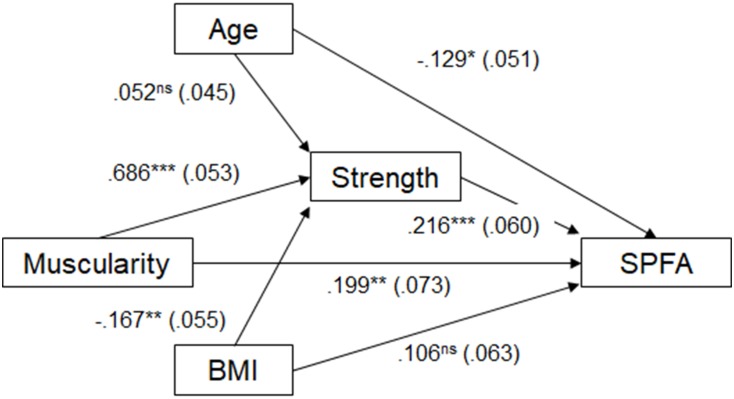
Unstandardized regression coefficients between upper body muscularity and self-perceived fighting ability mediated by strength and statistically controlling for BMI. The standard errors are shown in parentheses. ^∗^*p* < 0.05, ^∗∗^*p* < 0.01, ^∗∗∗^*p* < 0.001, ns, non-significant.

For the second prediction, the linear regression model indicated a positive relationship between upper body muscularity, while controlling for BMI and age, and self-perceived fighting ability [*R*^2^ = 0.14, *F*_(3,357)_ = 21.121, *p* < 0.001]. Accordingly, self-perceived fighting ability increases with greater muscularity (*B* = 0.411, *SE* = 0.073, β = 0.346, *p* < 0.001; see Figure 2). We conducted a mediation analysis of the second part of the prediction. We tested a simple mediation model in which strength mediates the relationship between upper-body muscularity and self-perception of fighting ability, with BMI and age included as covariates. The results showed a simple mediation, since results from bootstrapping yielded a significant indirect effect of upper-body muscularity on self-perceived fighting ability based on strength (*B* = 0.168, BootSE = 0.051, β = 0.148), with a 95% bootstrapped confidence interval from 0.067 to 0.268 for the unstandardized coefficient (see Figure 3). The mediation was partial since the direct effect of muscularity on self-perceived fighting ability was still significant (*B* = 0.236, *SE* = 0.087, β = 0.199, *p* = 0.007), but lower than the effect when strength was not considered (*B* = 0.411, *SE* = 0.073, β = 0.346, *p* < 0.001).

## Discussion

The faculty to recognize visual cues of fighting ability in competitors is thought to be an important characteristic that allows men to avoid the costs of contest competition, especially when there is clear asymmetry between contestants (Maynard-Smith and Parker, 1976; Sell et al., 2009a). However, in order to assess this asymmetry, individuals need to compare the fighting ability of their competitor with their own. Research into this ability in human beings, strength, based on HGS measurements, has always been used as a reliable expression of muscularity and a proxy for fighting ability (Sell et al., 2009a, 2012; Muñoz-Reyes et al., 2015; Durkee et al., 2018). In this study, we tested two main predictions, the first about the relationship between upper-body muscularity and strength, and the second about the role of muscularity in the self-perception of fighting ability, with this effect being mediated by strength. Our results confirmed both hypotheses: we found a positive relationship between upper-body muscularity and strength, and we showed that upper-body muscularity relates positively to the self-perception of fighting ability, with this effect being partially mediated by strength. However, our results suggest that the robustness of the relationship between strength and upper-body muscularity has been overestimated, and that there are more traits that influence the self-perception of fighting ability.

Following the first prediction, it is commonly assumed that muscularity is an easily observable trait that can be used as a cue of strength when evaluating the fighting ability of a potential rival (Durkee et al., 2018). This argument assumes that muscularity and strength are strongly related. In agreement, we found the relationship as expected, as more muscular men are stronger than less muscular men. However, upper-body muscularity only explained 36% of the variance in strength. Other works have found similar degrees of variance in the relationship between muscularity and strength, indicating that although the two variables are related, there is a significant percentage of variance in strength that cannot be explained by muscle mass (Johnson et al., 1994). In this sense, the muscle “efficiency,” i.e., muscle strength per unit of muscle mass, may be determined by a number of factors, such as weight at birth (Kuh et al., 2002), the number and type of fibers in the muscle (Folland and Williams, 2007) and neural adaptations (Häkkinen et al., 1998; Folland and Williams, 2007). These factors may result in individuals with similar muscle mass having different degrees of strength, which weakens the relationship between muscularity and strength. However, another explanation for the low relationship between muscularity and strength centers on the specific method used for measuring strength. Although HGS is considered a good proxy for upper body strength (e.g., Sell et al., 2009a; Wind et al., 2010), and it has been proved to be a reliable indicator of health and fitness in men (Gallup and Fink, 2018), it involves a limited group of muscles measured under static conditions. Therefore, HGS may be a measurement of upper body strength that reflect only a part of capabilities of individuals.

Regarding our second prediction about the self-perception of fighting ability, individuals have their personal experience and history of interactions as a basis for aligning their strength in function of their fighting ability. Thus, if the critical trait in determining fighting ability were strength, muscularity would be related to self-perception of fighting ability, but the effect would be mediated by strength. We found partial support for this argument, since upper body muscularity is related to self-perception of fighting ability in our sample. However, its effect was only partially mediated by strength, suggesting that individuals rely on their perception of their own muscle mass when assessing fighting ability. In other words, more muscular men perceive their fighting ability as greater, not only because they are stronger (at least in the HGS task). This finding raises the question about what benefits other than strength muscle mass provides in a contest. First, muscle mass of the upper-body may function as an intimidation signal for potential rivals. Therefore, more muscular men would perceive their abilities in fights as being greater because rivals tend to avoid direct confrontation in view of their muscularity, regardless of their real strength (Frederick et al., 2007). This argument makes sense from an adaptive perspective. There is in fact comparative (Manson and Wrangham, 1991) fossil (Lahr et al., 2016) and experimental evidence, the latter derived from economic games (Navarrete et al., 2010), that indicates that intergroup conflict was a major source of selection pressure, especially for the development of aggressive and formidability traits in human male ancestors. Therefore, muscularity could be a powerful cue, functioning as an intimidation signal, in scenarios of intergroup conflicts between males that have not interacted previously.

Together, our results underline the need to consider that upper body muscularity may be related to other sources of strength than hand-grip or other benefits in fights. Hand-grip strength is an isometric force task that is static and involves only the activation of the muscles of the forearm. Studies have shown that HGS is less sensitive to loss of fat free mass than dynamic strength tasks like the clean lift and endurance tasks (Johnson et al., 1994). The clean lift task not only adds the dynamic dimension, but also involves more muscles. Both coordinated movement and endurance are likely important in fighting, especially in order to maximize physical costs through precise hits, and to withstand extended conflicts. Accordingly, upper body muscularity may improve fighting ability by increasing movement coordination and endurance, in addition to its effect on strength.

The major limitation of our study is that only measuring maximum HGS constrained us to investigate what benefits greater upper-body muscularity can provide in fights other than HGS. Future studies should test if muscularity measured by bioelectrical impedance is related to endurance and dynamic strength exercises. In addition, Sell et al. (2012) proposed several sexually dimorphic physical and psychological characteristics that contribute to fighting ability, such as the ability to dissipate heat, stronger bones, more accurate blocking of thrown objects, faster mental rotation and spatial visualization, etc. Future studies should include these psychological and physical characteristics to determine their effects on self-perception of fighting ability and their interaction with muscularity and strength. Finally, our result also indicates that positive relationship between muscularity and attractive, could be sustained in the possibility of muscularity being a reliable indicator of a major group of honest characteristics other than strength. However, has to be done future studies in this area.

To conclude, our study sheds light on the role of muscular mass and strength in the self-perception of fighting ability. We found that in spite of the prevailing use of muscular mass as a visual cue of our opponent’s strength and thus of their fighting ability much of the variance in strength is not explained by upper body muscular mass, and the effect of upper body muscular mass on self-perception of fighting ability was only partially mediated by strength. However, alternative explanations are possible, and we need to consider other variables jointly with HGS to estimate upper body strength and thus of fighting ability. Taking into account this limitation, together, our results suggest that upper body muscularity has direct benefits in contest competition other due to: (i) possible links between upper body muscularity and fighting ability not mediated by strength; and (ii) the value of the signal itself as an intimidation trait, especially in contexts where fighting contests are less recurrent and thus there is less public information about individuals fighting ability.

## Author Contributions

JM-R and PPo conceived the study. JM-R, PPo, PPa, NV, and OR collected the data. JM-R, PPo, and CR-S contributed to data analysis and drafted the manuscript. All authors editing the manuscript for intellectual content, provided critical comments on the manuscript, and agreed to be accountable for the content of the work.

## Conflict of Interest Statement

The authors declare that the research was conducted in the absence of any commercial or financial relationships that could be construed as a potential conflict of interest.

## Footnotes

1 www.labcah.cl

2 http://www.afhayes.com
